# Ferroptosis: A new insight for treatment of acute kidney injury

**DOI:** 10.3389/fphar.2022.1065867

**Published:** 2022-11-17

**Authors:** Shiyang Li, Rui Wang, Yixue Wang, Yong Liu, Yingjin Qiao, Peipei Li, Jingfang Chen, Shaokang Pan, Qi Feng, Zhangsuo Liu, Dongwei Liu

**Affiliations:** ^1^ Traditional Chinese Medicine Integrated Department of Nephrology, The First Affiliated Hospital of Zhengzhou University, Zhengzhou, China; ^2^ Research Institute of Nephrology, The First Affiliated Hospital of Zhengzhou University, Zhengzhou University, Zhengzhou, China; ^3^ Henan Province Research Center for Kidney Disease, Zhengzhou, China; ^4^ Key Laboratory of Precision Diagnosis and Treatment for Chronic Kidney Disease in Henan Province, Zhengzhou, China; ^5^ Blood Purification Center, The First Affiliated Hospital of Zhengzhou University, Zhengzhou, China

**Keywords:** acute kidney injury, ferroptosis, mechanisms, regulators, treatment

## Abstract

Acute kidney injury (AKI), one of the most prevalent clinical diseases with a high incidence rate worldwide, is characterized by a rapid deterioration of renal function and further triggers the accumulation of metabolic waste and toxins, leading to complications and dysfunction of other organs. Multiple pathogenic factors, such as rhabdomyolysis, infection, nephrotoxic medications, and ischemia-reperfusion injury, contribute to the onset and progression of AKI. However, the detailed mechanism remains unclear. Ferroptosis, a recently identified mechanism of nonapoptotic cell death, is iron-dependent and caused by lipid peroxide accumulation in cells. A variety of studies have demonstrated that ferroptosis plays a significant role in AKI development, in contrast to other forms of cell death, such as apoptosis, necroptosis, and pyroptosis. In this review, we systemically summarized the definition, primary biochemical mechanisms, key regulators and associated pharmacological research progress of ferroptosis in AKI. We further discussed its therapeutic potential for the prevention of AKI, in the hope of providing a useful reference for further basic and clinical studies.

## Introduction

Acute kidney injury (AKI) is one of the most common and severe clinical kidney syndromes, with higher morbidity and mortality globally. Epidemiological investigations showed that the morbidity of AKI is over 20% in adults and over 30% in children, and AKI-triggered mortality has reached up to 23.9% in adults (Susantitaphong et al., 2013). The high mortality, expense, and potential for progression to chronic kidney disease (CKD) make AKI a major worldwide health issue and research priority (Mehta et al., 2015). A growing body of evidence has demonstrated that multiple pathogenic factors, including rhabdomyolysis, infection, nephrotoxic drugs, and ischemia-reperfusion injury, contribute to the initiation and progression of AKI (Basile et al., 2012). The current therapeutic methods for AKI mainly include correcting acid-base balance and electrolyte disorder, correcting volume load, avoiding nephrotoxic drugs, and renal replacement therapy (Levey and James, 2017). However, the specific mechanism linked to the occurrence and development of AKI has yet to be identified. There are currently no effective treatment methods to prevent the onset of AKI, delay its progression, and promote its repair. Therefore, it is necessary to deeply investigate the specific pathogenesis of AKI and develop new corresponding therapies for clinical treatment. The main pathological manifestations of AKI are cell injury, apoptosis and renal tubular epithelial cell abscission. Recent studies have shown that iron-overload induced ferroptosis of renal tubular epithelial cells is positively correlated with the incidence rate and mortality of clinical AKI (Feng et al., 2022). There is direct evidence that iron chelating agents and small molecular ferroptosis inhibitors possess renal protective effects among various animal models of AKI, suggesting that ferroptosis plays an important role in the initiation and progression of AKI. Therefore, it is of great significance to explore the mechanism of ferroptosis of renal tubular epithelial cells in AKI and clarify the effects of ferroptosis on the progression of AKI to CKD.

As early as in 2003, Dolma et al. found a new small molecule compound erastin, which can induce the death of RAS-mutant cancer cells (Dolma et al., 2003). However, this mode of cell death was different from what had been reported before, and this new type of cell death can be blocked by iron chelators and antioxidants, indicating that it is related to the accumulation of iron and active oxidation products in cells. Subsequently, similar to erastin, two new drugs RSL3 and RSL5 were also found to induce this pattern of cell death (Yagoda et al., 2007; Yang and Stockwell, 2008). Subsequently, deferoxamine and vitamin E were further proven to reduce and inhibit cell death. In 2012, Dixon et al. officially named this iron-dependent cell death mediated by excessive lipid peroxidation as ferroptosis for the first time. Intracellular iron retention, reduced glutathione (GSH) depletion and iron-dependent lipid reactive oxygen species (ROS) accumulation are the main characteristics of ferroptosis (Dixon et al., 2012). The excessive accumulation of ROS can activate intracellular oxidative stress response, damage proteins, nucleic acids and lipids, and ultimately result in the occurrence ferroptosis (Tang et al., 2021). In contrast, ferroptosis is different from the previously identified regulatory cell death patterns such as apoptosis, necrosis and autophagy in morphology, genetics and mechanism (Hirschhorn and Stockwell, 2019) (Table 1). Particularly, in the process of ferroptosis, it will not cause the chromatin concentration that occurs in apoptosis, destruction of capsule integrity that occurs in necrosis, and formation of bilayer vesicle autophagy bodies that occur in autophagy (Mou et al., 2019). Morphologically, the cell volume becomes smaller, rounder and separated from each other, the volume of mitochondria decreases, the membrane density increases, the cristae decrease or disappear, tremendous iron ions are distributed in mitochondria and endoplasmic reticulum, but the cell membrane maintains its integrity and the nucleus remains normal in size (Qiu et al., 2020). In addition, no characteristic manifestations of other regulatory cell death modes such as nuclear pyknosis, nuclear lysis and phagocytic vesicles were observed. Biochemically, it is mainly manifested in the depletion of intracellular GSH, the decrease of glutathione peroxidase 4 (GPX4) activity and the increase in iron ion levels, and Fe^2+^ oxidizes lipids in a Fenton reaction-like manner, resulting in lipid peroxidation and a large amount of ROS, which promotes ferroptosis (Li et al., 2020a). Genetically, ferroptosis is a biological process regulated by multiple genes, which mainly participate in genetic variance in iron homeostasis and lipid peroxidation metabolism, but the specific regulatory mechanism needs to be further investigated. Additionally, due to the accumulation of autophagosomes in response to ferroptosis activators (such as erastin and RSL3) and the involvement of autophagy machinery components (such as ATG3, ATG5, ATG4B, ATG7, ATG13, and BECN1), ferroptosis has recently been regarded as a type of autophagy-dependent cell death. NCOA4-facilitated ferritinophagy, RAB7A-dependent lipophagy, BECN1-mediated system Xc^−^ inhibition, STAT3-induced lysosomal membrane permeability, and HSP90-associated chaperone-mediated autophagy may all contribute to ferroptosis (Zhou et al., 2020).

**TABLE 1:** The comparative study of different types of cell death.

Type of cell death	Morphological features	Biochemical features	Regulatory genes	
Ferroptosis	Rupture of the cell membrane; reduction and morphological shrinkage of mitochondria; an increase in density of bilayer membrane structure; reduction or disappearance of mitochondrial cristae	Decrease in GPX4 activity; iron overload; depletion of glutathione; accumulation of ROS	GPX4, SLC7A11, Nrf2, p53, ACSL4, FSP1	Hirschhorn and Stockwell, (2019)
Autophagy	Autophagy body and autophagic lysosomes formation	Microautophagy is mediated by direct lysosomal engulfment of cytoplasmic cargo. Chaperone-mediated autophagy through heat shock proteins recognizes particular proteins and degrades in lysosomes. Macroautophagy is the fusion of autophagy bodies and lysosomes	RIP1, RIP3, PI3K, p53, ATG	Glick et al. (2010)
Apoptosis	Blebbing of the plasma membrane, cell rounding; decrease of cellular and nuclear volume; DNA cleavage; nuclear fragmentation, chromatin condensation; formation of apoptotic bodies	Mitochondrial outer membrane permeabilization through the intrinsic pathway, the extrinsic pathway, and the endoplasmic reticulum pathway	Caspases, Bcl-2 family proteins, TNF-α, Fas, p53	Wang et al. (2015); Bertheloot et al. (2021)
Necrosis	Increased cell membrane permeability, cell swelling; organelle deformation or swelling; plasma membrane rupture; chromatin condensation; cell component overflow	Decrease in ATP level through RIP3-MLKL related signaling pathways, PKC-MAPK-AP-1 related signaling pathway, ROS-related metabolic regulation pathway	PIPK3, ATG5, ATG7, Caspase-8, Beclin-1	Tonnus et al. (2019)
Pyroptosis	The cells swelled and expanded, and many bubbles like protrusions form; scorched bodies form; the cell membrane breaks and the content flows out	The formation of inflammatory bodies; the activation of inflammatory caspases gasdermin; the release of a mass of proinflammatory factors	Caspase-1/11, IL-18	Yu et al. (2021a); Bertheloot et al. (2021)
Cuproptosis	Reduction of mitochondria volume and cristae; increased density of bilayer membrane structure	Excessive Cu accumulation triggers disruption of iron-sulfur cofactors; stimulates harmful reactive oxygen species resulting from Fenton reactions; Cu-dependent, mitochondria-induced cell death	Lipoylation DLAT, PDHA1, PDHB, SLC25A3, FDX1, LIAS, HSP70	Wang et al. (2022a)

Many studies have reported that ferroptosis may act as a trigger of different pathological and physiological processes (Xie et al., 2016). Ferroptosis was recently demonstrated to play an important role in the development of various kidney diseases, including AKI (Feng et al., 2022). Before the identification of ferroptosis, apoptosis and necrosis were believed to be the primary mechanisms of AKI-induced kidney damage, and numerous pertinent studies have mainly focused on necrosis and apoptosis. With the deep studies of ferroptosis and AKI, several experiments have identified that ferroptosis participates in the development of AKI. This review aimed to summarize the key regulators, molecular mechanisms, and pharmacological progress of ferroptosis, and further expound on the role of ferroptosis in the development of AKI, in the hope of providing new ideas for the clinical treatment, and prevention of AKI.

## Key molecular mechanisms and regulators of ferroptosis

**FIGURE 1:**
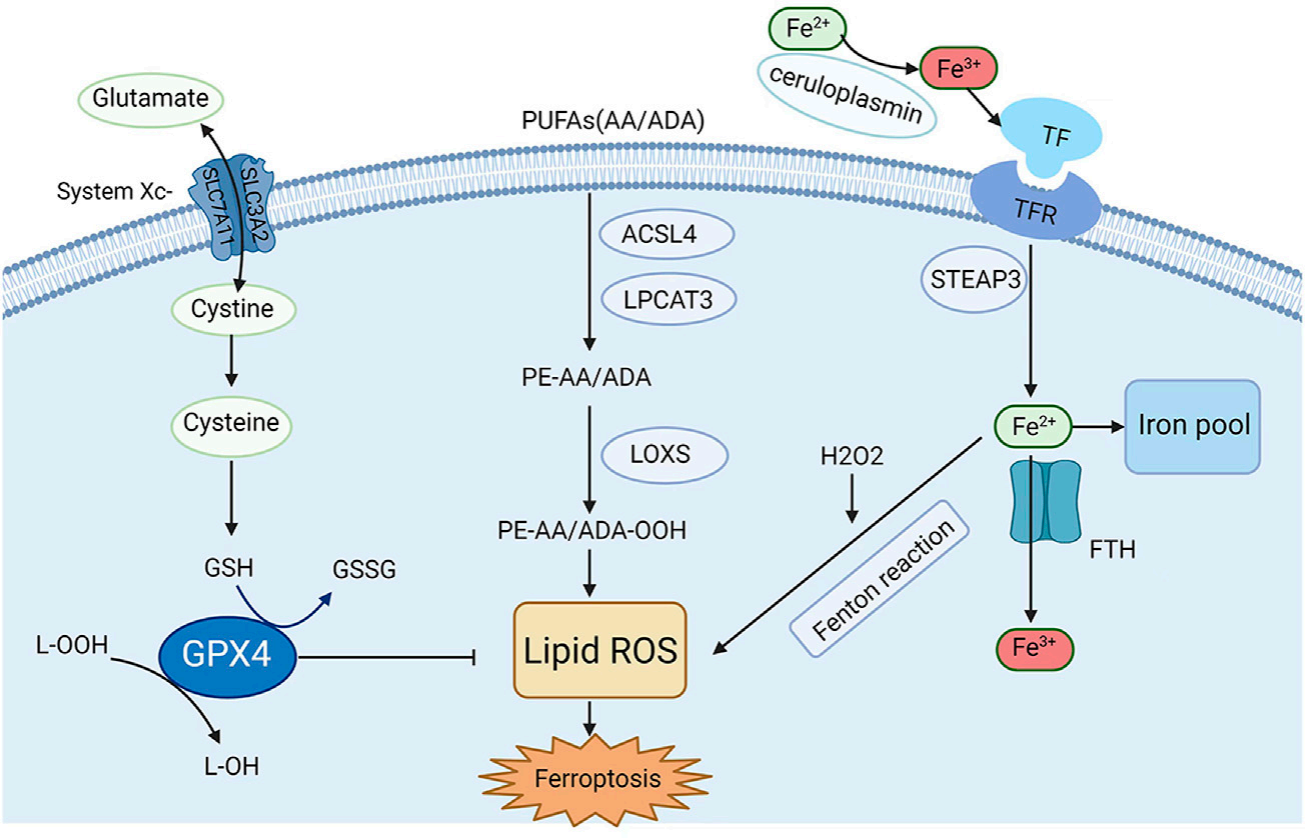
Mechanisms and key regulators of ferroptosis. Iron metabolism and lipid peroxidation are two key regulatory pathways for ferroptosis, and several key regulators (e.g., system Xc^−^, GPX4, and acyl-CoA synthetase long-chain family member 4 [ACSL4]) are also involved in ferroptosis.

Recently, the process and function of ferroptosis, as well as its impact on disease susceptibility, have been well explored. In addition, several regulators and molecular mechanisms of ferroptosis have been extensively investigated, such as system Xc^−^, GPX4, nuclear factor erythroid 2-related factor 2 (Nrf2), iron metabolism and lipid peroxidation. Herein, in this section, a briefly description of the key molecular mechanisms and regulators and related signaling pathways involved in ferroptosis are summarized in Figure 1.

## Iron metabolism

Iron is one of the most significant microelements in the human body, and its homeostasis is very important for the normal physiological function of cells (van Swelm et al., 2020). In general, Fe^2+^ from intestinal absorption or erythrocyte metabolism can be oxidized to Fe^3+^ by ceruloplasmin in plasma. This happens when it combines with transferrin (TF) to form the TF-TFR complex, and then ferroportin 1 (FPN1) on the cell membrane and moves into the cells by endocytosis. Ferritin is an intracellular iron-storing protein composed of ferritin heavy chain 1 (FTH1) and ferritin light chain (FTL), with FTH1 being active in iron oxidase, which can convert Fe^2+^ to Fe^3+^ (Cabantchik, 2014). The Fenton reaction produces abundant free radicals when cells are overloaded with Fe^2+^, which finally leads to lipid peroxidation and ferroptosis (Anderson and Frazer, 2017). Under some physiological and pathological conditions, cells susceptible to ferroptosis show up-regulation of TFR expression and down-regulation of ferritin (including FTH1 and FTL) expression, indicating that increasing iron uptake or reducing stored forms of iron can induce ferroptosis (Dixon and Stockwell, 2014). Some iron chelators are strongly associated with eliminating lipid peroxide free radicals, which may contribute to alleviating ferroptosis-related diseases, including AKI.

## Reactive oxygen species

ROS are unavoidably formed during redox reactions, which are essential for organisms. Under normal circumstances, cells closely monitor the ROS level. The imbalance of ROS homeostasis may result in the occurrence of a series of chronic diseases. High levels of ROS will induce cytotoxicity and tissue oxidative damage (Cheung and Vousden, 2022). A large number of studies have reported that ROS can alter cell signaling proteins and mediate the development of atherosclerosis, diabetes, uninhibited cell growth, neurodegeneration, inflammation, and aging (Zhang et al., 2016). In addition, ROS also participate in metabolic regulation and stress responses through the primary medium of specific target protein-mediated signaling pathways, which is beneficial for cells to adapt to changes in the environment and stress. Therefore, ROS homeostasis is essential for the body to maintain its function. Previous researches have demonstrated that the accumulation of ROS-induced lipid peroxidation is the ultimate course of ferroptosis (Yang and Stockwell, 2016). Two mechanisms are responsible for the accumulation of ROS during ferroptosis. The first mechanism is that the function of GPX4 is inhibited and cannot timely remove ROS produced by cell oxidation reaction. The other mechanism is that the excessive Fe^2+^ in cells generates a considerable amount of ROS through the Fenton reaction. Therefore, GPX4 inhibition and intracellular Fe^2+^ overload caused by ROS accumulation play key roles in regulating the initiation and progression of ferroptosis.

## Glutathione peroxidase 4

Glutathione peroxidases (GPXs) are members of the peroxidase enzyme family. Their primary biological role is the reduction of lipid hydroperoxide and free hydrogen peroxide to protect organisms from oxidative damage (Jiao et al., 2017). To date, eight sub-members of GPXs have been identified in the human body, in which GPX4 has been found to play a key role in ferroptosis. As an evolutionarily highly conserved and highly efficient reductase, GPX4 can convert toxic lipid hydroperoxides (L-OOH) into nontoxic lipid alcohols (L-OH) *via* an indispensable partner glutathione (GSH), therefore restricting the propagation of lipid peroxidation and the accumulation of lipid peroxides (Forcina and Dixon, 2019; Ursini and Maiorino, 2020). Studies have been around for decades showing that the morphology of cysteine-deprived cell death differed from that of other amino acids deprived cell deaths (Eagle, 1955). As a small molecule active peptide containing sulfhydryl group, GSH is composed of glutamic acid, cysteine, and glycine (Kalinina and Novichkova, 2021). Glycine is considered as the richest low-molecular-weight mercaptan compound synthesized in cells, and its sulfhydryl structure can be oxidized and dehydrogenated, allowing it to play an important role in antioxidation and free radical scavenging to play a central role in some metabolic pathways and cytoprotective mechanisms (Forman et al., 2009). GSH biosynthesis occurs in the cytoplasm and is present in the most vital cell regions, including the mitochondria, nucleus and endoplasmic reticulum (Scirè et al., 2019). Furthermore, GSH is a cofactor of GPX4 catalytic reaction. The consumption of GSH in cells significantly reduces the GPX4 activity, resulting in the accumulation of lipid peroxide and cell ferroptosis. (Ursini and Maiorino, 2020). Angeli et al. found that the inactivation of GPX4 can result in ferroptosis-related cell death, which leads to AKI in mice (Friedmann Angeli et al., 2014). Recent studies have found that autophagy activation mediated by acid sphingomyelinase (ASM), one of the key enzymes in sphingolipid metabolism, is essential for GPX4 degradation and ferroptosis activation (Thayyullathil et al., 2021). The function of GPX4 is the core of ferroptosis, and its significance has been gradually uncovered. Inhibition of GPX4 expression or hindering its function may cause oxidative damage to cells or tissues. Many studies have shown that the antioxidant function of GPX4 is the central link to ferroptosis (Ursini and Maiorino, 2020). Therefore, the molecules that affect the function and activity of GPX4 may significantly affect the incidence of ferroptosis.

## System Xc^-^

System Xc^−^ is an amino acid transporter widely expressed on the cell membrane, a channel for cystine and glutamate, and a heterodimer composed of SLC7A11 and SLC3A2. Intracellular and extracellular cystine and glutamate are exchanged in a 1:1 ratio, and their concentrations affect the transport function (Dixon et al., 2014; Ursini and Maiorino, 2020). Cystine enters the cell through system Xc^−^ exchange with glutamate, rapidly reduces to cysteine, and subsequently contributes to the synthesis of GSH. Thus, promoting the function of System Xc^−^ can inhibit ferroptosis. According to recent studies, the uptake of cystine mediated by SLC7A11 promotes GSH and GPX4 protein synthesis, and ultimately inhibits ferroptosis (Zhang et al., 2021a). Moreover, polydatin could dose-dependently alleviate cell death induced by the system Xc^−^ inhibitor (Zhou et al., 2022). Another study indicated that downregulation of connexin 43 (Cx43) can inhibit ferroptosis by restoring the level of SLC7A11to alleviate cisplatin-induced AKI (Yu et al., 2021b). In turn, inhibition of System Xc^−^ promotes the occurrence and development of ferroptosis. For example, Patulin (PAT), a common food-borne mycotoxin, decreased activity of SLC7A11 by activating AMP-activated protein kinase (AMPK)-mediated formation of the beclin1-SLC7A11 complex and exacerbated folic acid-induced nephrotoxicity in a mouse model of AKI (Chen et al., 2022). Research has shown that targeted inhibition of the SLC3A2 subunit in system XC^−^ can induce ferroptosis (Ma et al., 2021a). Therefore, maintaining the normal function of system Xc^−^ is crucial to protecting cells and tissues from ferroptosis.

## Polyunsaturated fatty acids

Lipid metabolism-related ferroptosis is closely related to AKI, and the existing research found that changes in lipid metabolism can drive AKI (Bugarski et al., 2021). Many studies have reported obvious fatty acid oxidation dysfunction and lipid deposition in cisplatin-induced AKI (Nagothu et al., 2005). Another study showed that ω3-polyunsaturated fatty acids (ω3-PUFAs) can attenuate Ischemia-reperfusion (I/R) injury-induced AKI in fat-1 mice (Gwon et al., 2017). The carbon-carbon double bonds present in PUFAs are unstable; when compared to saturated PUFAs, PUFAs are easily oxidized to lipid peroxides (Yang et al., 2016). The peroxidation of PUFAs in lipid membranes is the core and concluding step of ferroptosis. Arachidonoyl (AA) and adrenic acid (ADA) are the two most specific lipid membrane PUFAs in ferroptosis. The peroxidation of PUFAs is driven by acyl-CoA synthase long-chain family member 4 (ACSL4). AA and ADA are esterified into acyl CoA derivatives by ACSL4, esterified into phosphatidyl ethanolamine (PE-AA/ADA) by lysophosphatidylcholine acyltransferase 3 (LPCAT3), and then oxidized into lipid peroxides (PE-AA/ADA-OOH) by lipoxygenases (LOXs). Additionally, excessive Fe^2+^ can react with lipid peroxides to generate free radicals, further propagating lipid peroxidation. Mishima et al. proved that some drugs that scavenge lipid peroxyl radicals can help control ferroptosis-related disorders, including AKI (Mishima et al., 2020). Similarly, cholesterol-rich membrane structures will also undergo peroxidation (Zielinski and Pratt, 2016). The antioxidant system continuously reduces lipid peroxides produced in the normal oxidation environment to form a balance. If the antioxidant system is damaged, excessive activation of ACSL4, LPCAT3, and LOXs, and excessive Fe^2+^ can lead to high consumption of cell membrane phospholipids, and the accumulation of lipid peroxides will result in ferroptosis (Kagan et al., 2017; Ursini and Maiorino, 2020).

## Ferroptosis suppressor protein 1

Ferroptosis suppressor protein 1 (FSP1) was previously known as apoptosis-inducing factor mitochondrion-associated 2 (AIFM2). Sebastian et al. identified and suggested in 2019 that FSP1 and coenzyme Q10 (CoQ10) inhibited ferroptosis. Reduced CoQ10 could capture lipid peroxyl radicals in the cytoplasm, thereby preventing lipid peroxidation of the phospholipid membrane and inhibiting ferroptosis. Meanwhile, FSP1 can catalyze the regeneration of CoQ10 in the presence of NAD(P)H (Doll et al., 2019). Together with the GPX4-related pathway, this pathway inhibits lipid peroxidation and ferroptosis (Bersuker et al., 2019).

## Nuclear factor erythroid 2-related factor 2

Nrf2 is a transcription factor that is able to bind nuclear factors and coordinatethe basal and stress-induced activation of many cytoprotective genes, especially genes that counteract oxidative and electrophilic stresses (Itoh et al., 1997; Tonelli et al., 2018). Normally, Nrf2 is kept basally low by three different E3-ubiquitin ligase complexes: Kelch-like ECH-associated protein 1-Cullin 3-Ring box 1 (KEAP1-CUL3- RBX1), S-phase kinase-associated protein 1-Cullin1-Rbx1/β-transducin repeat-containing protein (SCF/β-TrCP), and synoviolin/Hrd1 (Dodson et al., 2019). In response to different activating stimuli, Nrf2 transfers to the nucleus, where it initiates the transcription of antioxidant response element (ARE)-containing genes (Nguyen et al., 2009). Interestingly numerous ferroptosis-related proteins and enzymes, such as GPX4, system Xc^−^ subunit, and GSH synthase, are Nrf2 target genes (Rojo de la Vega et al., 2018; Tonelli et al., 2018). Moreover, Nrf2 targets play an important role in iron metabolism, and Nrf2 is responsible for several genes involved in hemolysin, hemoglobin catabolism, iron storage, and iron export (Kerins and Ooi, 2018). To maintain intracellular iron homeostasis, the activation of Nrf2 can up-regulate the expression of iron metabolism-related proteins, such as ferritin (light chain and heavy chain), intracellular iron storage proteins, iron chelatase, etc. In conclusion, the role of Nrf2 in ferroptosis can be divided into three categories: iron metabolism, intermediate metabolism, and glutathione metabolism (Dodson et al., 2019). The specific target genes of Nrf2 are shown in Table 2. Multiple studies have confirmed that genetic or pharmacological enhancement of Nrf2 activity in the renal tubules significantly ameliorated damage related to AKI and that targeting the Keap1-Nrf2 system could prevent kidney disease progression (Nezu et al., 2017). A study showed that cordyceps cicadae mycelia could ameliorate cisplatin-induced AKI, and one of the mechanisms was through activating the HO-1/Nrf2 (Deng et al., 2020). Irisin could also protect against sepsis-induced AKI by activating the Nrf2 signaling pathway (Qiongyue et al., 2022). In conclusion, Nrf2 is closely related to ferroptosis and AKI.

**TABLE 2:** Target genes of Nrf2 involved in preventing ferroptosis.

	Target genes	Effects	References
GSH Metabolism	Glutamate-cysteine ligase modifier subunit (GCLM)	Promote GSH synthesis	Sasaki et al. (2002)
	GSH synthetase (GSS)	Promote GSH synthesis	Sasaki et al. (2002)
	GPX4	Anti-lipid peroxidation	(Rojo de la Vega et al., 2018; Tonelli et al., 2018)
	NADPH quinone dehydrogenase 1 (NQO1)	Anti-lipid peroxidation	Ross and Siegel, (2021)
	SLC7A11	Transport cysteine into cells	Fan et al. (2017)
	glutathione-S-transferases pi 1 and alpha 1 (GSTP1 and GSTA1)	Reduction of oxidized substrates with GSH or NADPH	Nebert and Vasiliou, (2004)
Intermediate metabolism	Small heterodimer partner (SHP)	Involve in lipid metabolism	Huang et al. (2010)
	Peroxisome proliferator-activated receptor γ (PPARG)	Involve in lipid metabolism	Cho et al. (2010)
	Aldo-ketoreductases (AKR1B1, AKR1B10)	Reduction of aldehydes and ketones to alcohols	Jung et al. (2013)
	Glucose-6-phosphate dehydrogenase (G6PD)	Glucose metabolism/NAPDH regeneration	Thimmulappa et al. (2002)
	Aldehyde dehydrogenase 1 family member A1 (ALDH1A1)	Oxidation of aldehydes to carboxylic acids	Duong et al. (2017)
Iron metabolism	ABCB6	Participate in the heme biosynthetic pathway	Campbell et al. (2013)
	Ferrochelatase (FECH)	Participate in the heme biosynthetic pathway	(Campbell et al., 2013; Yang et al., 2022a)
	Heme oxygenase-1 (HO-1)	Decompose heme into biliverdin and free iron	Alam et al. (1999)
	Biliverdin reductase A (BLVRA)	Repress BACH1 and allows Nrf2 to express HO-1	(Klóska et al., 2019; Consoli et al., 2021)
	Ferritin heavy chain 1 (FTH1)	Subunit of transferrin that affects iron ion transport	Tsuji et al. (2000)
	Ferritin light chain (FTL)	Subunit of transferrin that affects iron ion transport	Tsuji et al. (2000)
	Ferroportin 1 (FPN1)	Unstable iron can be exported from the cytosol through FPN1	Pietsch et al. (2003)

## Other regulators

Other regulators or pathways are also closely related to ferroptosis. Su et al. demonstrated that pannexin 1 (PANX1), a family protein deletion of the ATP release pathway, can regulate ferroptosis by activating the mitogen-activated protein kinases (MAPK)/extracellular signal-regulated kinase (ERK) pathway, thereby protecting the kidney from ischemia-reperfusion injury (Su et al., 2019a). The SLC39/ZIP family is a group of transmembrane transporters of divalent metal ions. The SLC39A14/ZIP14 and SLC39A8/ZIP8 transporters are closely associated with ferroptosis. Yu et al. found that conditional knockout of liver SLC39A14 could reduce liver iron accumulation and hepatic fibrosis mediated by ferroptosis, indicating that SLC39A14 can promote liver cell ferroptosis through mediated iron uptake (Yu et al., 2020). A study found that erastin-induced ferroptosis in neuronal cells was accompanied by BH3-interacting domain death agonist (BID) transactivation to mitochondria, loss of mitochondrial membrane potential, enhanced mitochondrial fragmentation and reduced ATP levels. Mitochondrial transactivation of BID links ferroptosis to mitochondrial damage (Neitemeier et al., 2017). Lee et al. found that inactivation of AMPK largely abolishes the protective effects of energy stress on ferroptosis *in vitro* and on ferroptosis-associated renal ischemia–reperfusion injury *in vivo* (Lee et al., 2020). Moreover, a study showed that hyperactive mutation of PI3K-AKT-mTOR signaling protects cancer cells from ferroptosis through SREBP1/SCD1-mediated lipogenesis (Yi et al., 2020). However, the knowledge of these pathways is insufficient, and we still need to find their internal relationship.

## Research progress of ferroptosis-related intervention reagents

Recently, many natural and synthetic drugs have been found to induce or inhibit ferroptosis by regulating related pathways, which has great therapeutic potential for ferroptosis-related diseases. In various proliferative diseases such as tumors, inducing ferroptosis can promote tumor cell death and play an anti-tumor role. In some non-neoplastic diseases (such as ischemia-reperfusion injury, cardiovascular and cerebrovascular diseases, kidney diseases, etc.), ferroptosis inhibitors can prevent the occurrence and development of lipid peroxidation and ferroptosis by different targets in the related pathway. Several ferroptosis-related inducers and inhibitors are shown in Table 3.

**TABLE 3:** Inducers and inhibitors of ferroptosis.

Classification	Reagents	Targets	References
Ferroptosis Inducers	Erastin	System Xc^−^, VDAC2/3, p53	(Dolma et al., 2003; Yang et al., 2020)
	Sulfasalazine	System Xc^-^	Gout et al. (2001)
	Sorafenib	Nrf2, Keap1, System Xc^-^	Louandre et al. (2015)
	ATF3	SLC7A11, GPX4	(Wang et al., 2020; Wang et al., 2021)
	Acetaminophen	GSH, GPX4	Mitchell et al. (1973)
	RSL3	NF-κB, GPX4	Yang et al. (2014)
	FINO2	GPX4, Fe^2+^	Gaschler et al. (2018)
	Lipopolysaccharide	SLC7A11, GPX4	Liu et al. (2020a)
	Legumain	GPX4	Chen et al. (2021)
	Dexamethasone	GSH	von Mässenhausen et al. (2022)
	DPI2	GSH	Yang et al. (2014)
	BSO	GSH	Yang et al. (2014)
	Cisplatin	GSH, GPX4	Yamaguchi et al. (2013)
	FIN56	GPX4, CoQ10	Shimada et al. (2016)
	Siramesine	FPN, Ferritin	Ma et al. (2016)
Ferroptosis inhibitors	Fer-1	Lipid peroxidation	Skouta et al. (2014)
	SRS 16–86	Lipid peroxidation	Skouta et al. (2014)
	SRS 11–92	Lipid peroxidation	Linkermann et al. (2014a)
	Liproxstatin-1	GPX4, Lipid peroxidation	Friedmann Angeli et al. (2014)
	Vitamin E	Lipid peroxidation	Hinman et al. (2018)
	Vitamin K	FSP1	Mishima et al. (2022)
	Quercetin (QCT)	MDA, ROS, GSH	Wang et al. (2021)
	Nuciferine	Lipid peroxidation	Li et al. (2021)
	Irisin	GPX4, Lipid peroxidation	Zhang et al. (2021b)
	VDR	GPX4, Lipid peroxidation	Hu et al. (2020)
	Ginsenoside Rg1	GPX4, GSH, FSP1	Guo et al. (2022)
	Entacapone	Nrf2	Yang et al. (2022b)
	MCTR1	Nrf2	Xiao et al. (2021)

## Interplay between ferroptosis and other types of cellular processes

It is generally believed that the main pathological characteristic of AKI is damage and death of renal tubules. In the past few decades, several cellular processes have been reported in AKI, such as apoptosis, necrosis, autophagy and inflammation. Although ferroptosis has been confirmed to be a new form of cell death that differs from other cellular processes, recent studies have found that these cellular processes may coincide and be related in some ways. At the cellular level, all of these cellular processes are involved in mitochondrial alterations. Mitochondrial fragmentation, cytochrome c release and caspase activation lead to apoptosis in AKI induced by ischemia and cisplatin (Brooks et al., 2009). A range of mitochondrial dysfunction also occurs in necrosis (Vandenabeele et al., 2010). Moreover, current studies suggest that the diverse roles of mitochondria in bioenergetic, biosynthetic, and ROS regulation contribute to ferroptosis (Brenner et al., 2000; Gan, 2021). At the molecular level, excessive ROS attack biological membranes and propagate the lipid peroxidation chain reaction, which in turn induces different types of cell death, including apoptosis, autophagy and ferroptosis (Su et al., 2019b). In conclusion, we hypothesize that different cellular processes may coexist in AKI, but which type is dominant during the development of AKI and whether different cellular processes interact with each other need to be further studied.

## Ferroptosis in different types of acute kidney injury

### Rhabdomyolysis-induced acute kidney injury

When ferritin gene knockout mice develop AKI induced by rhabdomyolysis (RM), the mortality of mice increases, and the structural and functional renal injury is exacerbated (Zarjou et al., 2013), indicating that FTH plays an important role in AKI renal injury. Zorova et al. found that the levels of heme and free iron in renal cell cytoplasm and mitochondria were significantly increased in RM rats, as well as the level of lipid peroxide in renal tissue (Zorova et al., 2016). Guerrero-Hue et al. found that although apoptosis and necrosis were also activated, ferroptosis played a pivotal role in RM-induced AKI. Curcumin was also found to inhibit the toll-like receptor 4 (TLR4)/nuclear factor kappa-B (NF-κB) axis and activate HO-1 to reduce inflammation and oxidative stress, inhibiting ferroptosis and effectively improving renal function (Guerrero-Hue et al., 2019). The latest research by Zhao et al. showed that iron deficiency aggravates RM-induced AKI by increasing catalytic heme iron and that correcting iron deficiency may reduce all types of AKI (Zhao et al., 2021). The above studies suggested that ferroptosis may be involved in the occurrence and development of RM-induced AKI.

### Ischemia/reperfusion injury-induced acute kidney injury

Studies reported that ischemia/reperfusion injury (IRI)-induced AKI-associated ferroptosis is mediated by the mitochondrial cytokine augmenter of liver regeneration (ALR), which is related to the GPX4 system. Silencing the ALR gene leads to aggravation of mitochondrial damage, decreases GPX4 activity, and promotes ferroptosis (Huang et al., 2019). Another study also suggested that ferroptosis plays an important role in IRI-induced AKI, and through the inhibition of inositol requiring enzyme 1 (IRE1)/c-Jun NH_2_-terminal kinases (JNK) pathway can protect against IRI induced renal injury by inhibiting ferroptosis (Liang et al., 2022). Single-cell RNA sequencing (scRNA-seq) revealed that the ferroptosis-associated genes are mainly expressed in the renal tubular epithelium after IRI, but there is little expression of necroptosis and apoptosis-associated genes (Zhao et al., 2020). XJB-5-131 is an oxidation-resistant mitochondrial nitrogen oxide that reduces inflammatory cell infiltration, ferroptosis, and renal injury. XJB-5-131 showed excellent plasma stability, rapid plasma kidney transfer, and high renal affinity in mice. It is expected to be an effective drug for treating renal cell ferroptosis (Zhao et al., 2020). Ding et al. showed that miR-182-5p and miR-378a-3p are up-regulated in kidney IRI. By direct binding of GPX4 and SLC7A11, mRNA 3′UTR, GPX4, and SLC7A11 were negatively regulated, and ferroptosis was induced. Silencing this gene is expected to reduce the renal injury caused by ferroptosis (Ding et al., 2020). Moreover, Su et al. demonstrated that silencing the expression of pannexin1 can significantly prevent renal IRI by inhibiting MAPK/ERK activation in the ferroptosis pathway (Su et al., 2019a). By up-regulating GPX4, irisin treatment can reduce AKI caused by IRI. Treatment with the GPX4 inhibitor RSL3 eliminates the protective effect of irisin (Zhang et al., 2021b). Melatonin and entacapone were confirmed to upregulate the nuclear translocation of Nrf2, resulting in increased expression of the downstream SLC7A11 and significant suppression of oxidative stress and ferroptosis (Yang et al., 2022b; Huang et al., 2022). Recent research revealed that dexmedetomidine could attenuate ferroptosis-mediated IRI-induced AKI by inhibiting ACSL4 (Tao et al., 2022), and ACSL4 knockout also significantly alleviated this injury (Wang et al., 2022b). Ubiquitin specific peptidase 7 (USP7) inhibition attenuated IRI-induced renal injury by inhibiting ferroptosis through decreasing ubiquitination of TANK-binding kinase 1 (TBK1) and promoting DNMT1-mediated methylation of FMR1 (Dong et al., 2022). Michael et al. conducted a druggability screen to identify ferroptosis as a key driver pathway for maladaptive repair and found that pharmacological inhibition of ferroptosis ameliorates maladaptive kidney responses and fibrosis after severe IRI (Balzer et al., 2022). Various studies have shown that iron chelators and other ferroptosis inhibitors can reduce the renal damage induced by ischemia-reperfusion and that the inactivation of regulatory factors related to ferroptosis can cause or aggravate AKI (Friedmann Angeli et al., 2014; Linkermann, 2016). The above studies revealed ferroptosis plays an important role in AKI and silencing specific genes or proteins may provide specific potential targets for treating AKI.

### Folic acid-induced acute kidney injury

Low doses of folic acid (FA) are beneficial against oxidative stress (Hwang et al., 2011), while high doses of FA are widely used in the induction of animal kidney disease. As folate reduction by dihydrofolate reductase to form tetrahydrofolate uses large amounts of NADPH as a reducing power, high levels of folate in the kidneys can severely compromise cellular antioxidative systems, leading to aggravated redox imbalance and oxidative stress (Gupta et al., 2012; Ducker and Rabinowitz, 2017). Sanchez et al. found that in the AKI model induced by FA, lipid peroxidation and down-regulation of GSH metabolism occur in the kidney, a characteristic of ferroptosis (Martin-Sanchez et al., 2017). The ferroptosis inhibitor Fer-1 can prevent renal injury, reduce inflammation, and prevent the down-regulation of Klotho expression. However, inhibition of necrosis or apoptosis at the pharmacological or genetic level cannot achieve the above objectives, suggesting that ferroptosis is the main pathway of AKI injury induced by FA (Martin-Sanchez et al., 2017). Guo et al. also confirmed that ferroptosis is the main cause of FA-induced AKI, and targeted inhibition of Rev-erb-α/β can increase the transcription of SLC7A11 and HO-1, thereby inhibiting ferroptosis to improve FA-induced AKI (Guo et al., 2021). The latest research found that legumain, a conserved asparagine endopeptidase, promotes ferroptosis by enhancing GPX4 lysosomal autophagy in FA-induced AKI. Legumain deficiency can alleviate GPX4 lysosomal autophagy and improve renal injury (Chen et al., 2021). Li et al. reported that the bioactive compound nuciferine inhibits the formation of iron and lipid peroxidation, alleviates FA-induced AKI in mice, and inhibits the ferroptosis (Li et al., 2021). Another research showed that FG-4592, an inhibitor of prolyl hydroxylase of hypoxia-inducible factor (HIF), can reduce ferroptosis in the early stage of FA-induced renal injury *via* Akt/glycogen synthase kinase-3 beta (GSK-3β)-mediated Nrf2 activation, thereby delaying fibrosis progression (Li et al., 2020b). Wang et al. found that the natural flavonoid QCT can increase GSH, SLC7A11 and GPX4 levels by reducing the expression of ATF3, inhibiting ferroptosis in many aspects (Wang et al., 2021). These studies contribute to a better understanding of the relationship between ferroptosis and AKI and provide suggestions for developing new treatment strategies for AKI.

### Cisplatin-induced acute kidney injury

Cisplatin is an inorganic platinum-based chemotherapeutic agent that is widely used in the treatment of a variety of solid malignant tumors (Kodama et al., 2014). After a single dose of cisplatin, approximately one-third of the patients develop nephrotoxicity (Lebwohl and Canetta, 1998). A recent study identified Dpep1 and Chmp1α as kidney disease genes, and at the molecular level, both genes are crucial regulators of ferroptosis. Chmp1α knockdown enhances ferroptosis through increased iron accumulation in cisplatin induced AKI, and Dpep1 knockdown ameliorates cisplatin-induced apoptosis and ferroptosis. Moreover, Dpep1 and Chmp1α levels are also strongly and negatively correlated in AKI models (Guan et al., 2021). Studies have shown that cisplatin-induced reduced glutathione consumption and glutathione peroxidase inactivation can cause ferroptosis and tumor resistance (Guo et al., 2018). We speculated that AKI renal cells exhibit a similar effect. Human or mouse proximal tubular epithelial cells treated with cisplatin showed significant cell death, which ferroptosis inhibitors could reduce. Overexpression of myo-inositol oxygenase (MIOX) systematically aggravates oxidative damage, including a decrease in GPX4 activity and the levels of nicotinamide adenine dinucleotide phosphate (NADPH) and GSH. Inhibiting MIOX expression inhibits ferroptosis (Dutta et al., 2017; Deng et al., 2019). Hu et al. also verified that ferroptosis played an important role in cisplatin-induced AKI. Similarly, they found that VDR activation may bind to the promoter of GPX4 and inhibit ferroptosis by increasing the GPX4 expression, thereby preventing cisplatin-induced renal injury (Hu et al., 2020). Rheb1, a molecular switch in renal tubular cells, inhibits ferroptosis in renal tubular cells by maintaining mitochondrial homeostasis and alleviates cisplatin-induced AKI (Lu et al., 2020). Moreover, farnesoid X receptor (FXR) was found to regulate the transcription of ferroptosis-related genes and has a protective effect against cisplatin-induced AKI (Kim et al., 2022). Prevention of drug-induced AKI by inhibiting ferroptosis may be a future research direction.

### Sepsis-induced acute kidney injury

Sepsis is defined as organ dysfunction resulting from the host’s deleterious response to infection. One of the most common organs affected is the kidney, resulting in sepsis-induced AKI (SA-AKI). ROS is a major contributor to sepsis. Studies have shown that the ferroptosis inhibitor Fer-1 significantly attenuates septic renal injury (Yao et al., 2022). A recent study also showed that irisin inhibits ferroptosis and improves renal function in septic mice by reducing ROS production, iron content, and MDA levels, increasing GSH levels and changing the expression of GPX4 and ACSl4 in kidney tissue (Qiongyue et al., 2022). This result was consistent with the results of Fer-1. Another study verified the role of ferroptosis in sepsis-induced AKI and found that MCTR1 effectively suppressed ferroptosis in SA-AKI by upregulating Nrf2 expression (Xiao et al., 2021). Melatonin treatment was proven to suppress ferroptosis and alleviate sepsis-induced AKI by upregulating the Nrf2/HO-1 pathway (Qiu et al., 2022). Another small molecule, ginsenoside Rg1, can improve the viability of HK-2 cells and reduce the accumulation of iron and lipid peroxidation. Its anti-ferroptosis activity is dependent on FSP1 (Guo et al., 2022).

### Other types of acute kidney injury

**FIGURE 2:**
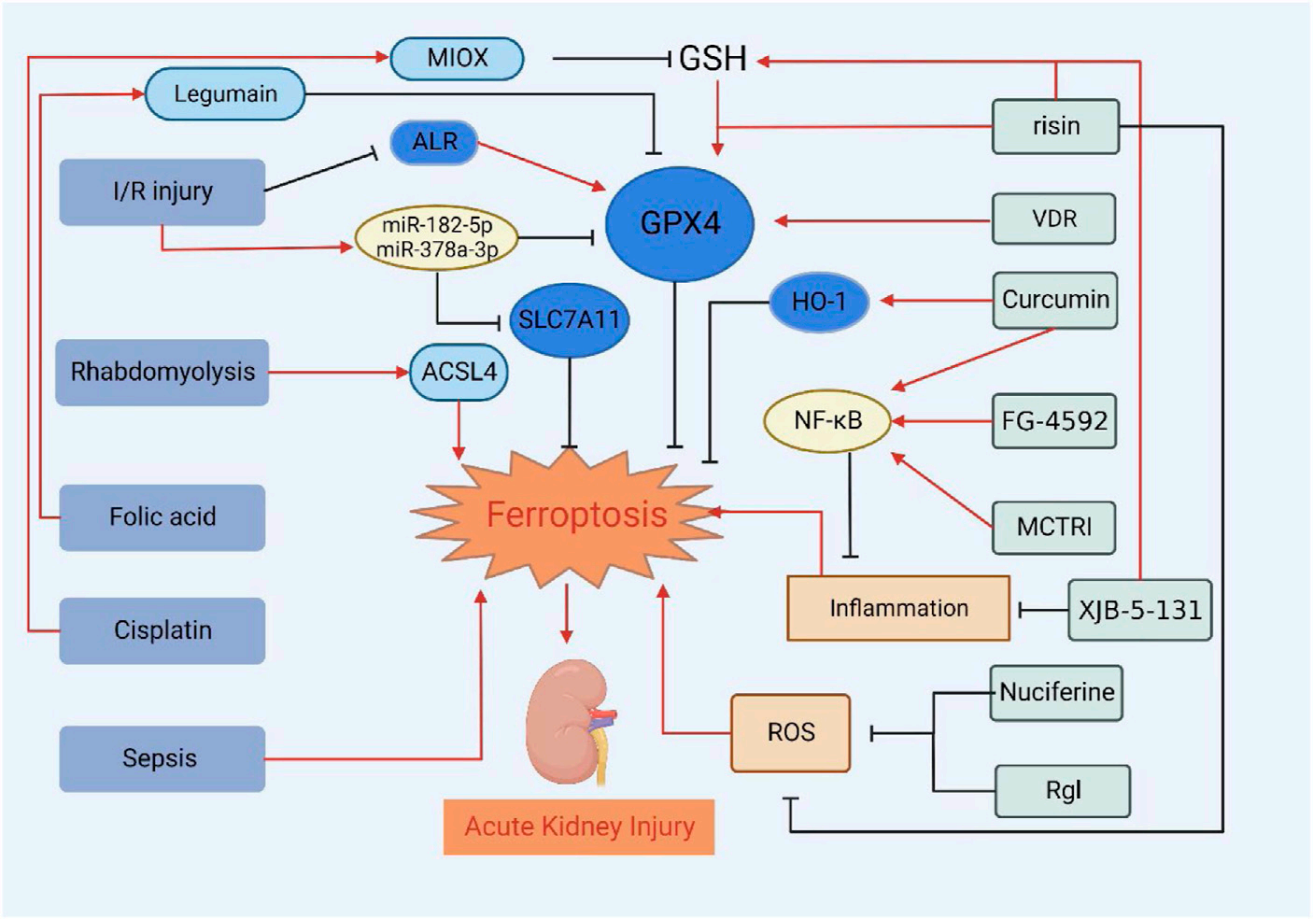
Treatment of AKI by targeting ferroptosis of renal tubular epithelial cells. VDR, risin, Curcumin, FG-4592, MCTR1, XJB-5-131, Nuciferine and RgI can alleviate or delay the development of AKI by inhibiting ferroptosis.

In addition, erastin is a ferroptosis inducer. Studies have demonstrated that erastin can also cause oxidative damage to renal tubular cells, which further confirms the role of ferroptosis in AKI (Hu et al., 2020; Chen et al., 2021). In AKI induced by other drugs such as gentamicin, glycerol, and cyclosporine, there are enhanced generation of reactive oxygen and hydrogen peroxide. Meanwhile, there are alterations in antioxidant defenses, such as GSH and HO-1. Scavengers of reactive oxygen metabolites as well as iron chelators can provide protection (Baliga et al., 1997). Severe acute pancreatitis (SAP)-induced AKI was followed by iron accumulation, increased lipid peroxidation, and upregulation of ferroptosis-related proteins and genes. Treatment with liproxstatin-1 alleviated pancreatic and renal histopathology injury in SAP rats (Ma et al., 2021b). There are many causes of AKI, and Figure 2 summarizes the current treatment progress of AKI by targeting ferroptosis of renal tubular epithelial cells. In addition to the common factors mentioned above, ureteral obstruction, surgery, other nephrotoxic drugs, etc. (Singh et al., 2012; Bao et al., 2018). More research is needed to determine the relationship between ferroptosis and AKI, but we speculate that regardless of the cause of AKI, the form of cell death is similar. Further studies can be performed to try to treat AKI by inhibiting ferroptosis.

## Discussion

AKI has always been a severe hazard to world health and the focus of research. In recent years, the morbidity and mortality of AKI have exhibited an increasing trend (Liu et al., 2020b), However, there is still no effective preventive and treatment methods. To maintain the stability of kidney function and protect human health, it is necessary to thoroughly understand the molecular mechanism of AKI and to develop appropriate treatment strategies to intervene and inhibit its further progression. Ferroptosis, rather than apoptosis and autophagy or other types of cell death has been identified as a new trigger for the occurrence of AKI (Martin-Sanchez et al., 2017). A study showed that genetic induction of high ferroptotic stress in proximal tubular (PT) cells after injury leads to the accumulation of inflammatory PT cells, enhancing inflammation, oxidative stress and fibrosis (Ide et al., 2021). This suggests an important role for ferroptosis in the progression from AKI to CKD. As mentioned above, several small molecules and drugs targeting the ferroptosis pathway have been shown to alleviate AKI in animal models. The identification of ferroptosis and the expansion of related research undoubtedly provide new ideas for the management of AKI.

In recent years, studies on ferroptosis have gradually increased in scope and depth. Many reports have revealed that ferroptosis is essential in the pathophysiology of various human diseases, including cancers, neurodegenerative diseases, cardiovascular diseases, liver diseases and kidney diseases. Several important molecules such as GPX4, FPN1, and SLC7A11, and genes such as Nrf2, have been shown to play a significant role in ferroptosis (Dixon et al., 2012; Tonelli et al., 2018); however, the complete pathways of ferroptosis from gene to protein to cell and tissue are still unclear. Even so its importance cannot be denied. Recent studies have gradually favored ferroptosis as the main form of cell death in AKI, but there is no doubt that other forms of cell death such as necrosis and apoptosis exist in AKI (Linkermann et al., 2014b). Moreover, ferroptosis, necrosis, and apoptosis overlap at the gene level (Hu et al., 2019). Future studies will focus on the specific pathways of ferroptosis, the interaction between each pathway, and the internal relationship between ferroptosis and other forms of cell death in AKI. This will benefit a deep understanding of the development process of AKI and the development of therapeutic targets covering a wider range. Although targeting ferroptosis has been shown to be effective in many animal experiments, animal models and humans are different, and more studies are needed to confirm that this strategy is also effective *in vivo*. Moreover, the extent to which ferroptosis can be used as a therapeutic target to restore AKI is the focus of future research. At present, known treatments related to ferroptosis are mainly applied in cancer treatment. Whether relieving AKI by ferroptosis inhibition will affect the occurrence and development of tumors is a challenge for future research. This reminds us whether alleviating AKI by inhibiting ferroptosis may have side effects on other systems. Therefore, further comprehensive investigation of ferroptosis in AKI development is urgently required to expand our knowledge and prevent kidney injury to benefit future clinical applications.

This review summarizes the recent scientific developments on AKI and ferroptosis, the close association between AKI and ferroptosis caused by different etiologies, and the effects of ferroptosis-related regulators, mechanisms and inhibitors on the prognosis of AKI. Through further investigation and exploration of ferroptosis, new therapies to improve AKI are expected to be found.
